# Framework, principles and recommendations for utilising participatory methodologies in the co-creation and evaluation of public health interventions

**DOI:** 10.1186/s40900-018-0136-9

**Published:** 2019-01-09

**Authors:** Calum F. Leask, Marlene Sandlund, Dawn A. Skelton, Teatske M. Altenburg, Greet Cardon, Mai J. M. Chinapaw, Ilse De Bourdeaudhuij, Maite Verloigne, Sebastien F. M. Chastin, on behalf of the GrandStand, Safe Step and Teenage Girls on the Move Research Groups

**Affiliations:** 1 Glasgow Caledonian University, School of Health and Life Sciences, Institute of Applied Health Research, Glasgow, UK; 2 NHS Grampian, Health Intelligence Department, Aberdeen, UK; 3 Department of Community Medicine and Rehabilitation, Umea University, Umea, Sweden; 4 Department of Public and Occupational Health, EMGO Institute for Health and Care Research, VU University Medical Center, Amsterdam, The Netherlands; 5 Department of Movement and Sports Sciences, Faculty of Medicine and Health Sciences, Ghent University, Ghent, Belgium

**Keywords:** Tailored intervention, Co-creation, Public health, Participation, Reflective learning

## Abstract

**Plain English summary:**

**Background:** Society has to cope with a large burden of health issues. There is need to find solutions to prevent diseases and help individuals live healthier lifestyles. Individual needs and circumstances vary greatly and one size fit all solutions do not tend to work well. More tailored solutions centred on individuals’ needs and circumstances can be developed in collaboration with these individuals. This process, known as co-creation, has shown promise but it requires guiding principles to improve its effectiveness. The aim of this study was to identify a key set of principles and recommendations for co-creating public health interventions.

**Methods:** These principles were collaboratively developed through analysing a set of case studies targeting different health behaviours (such as reducing sitting and improving strength and balance) in different groups of people (such as adolescent schoolgirls and older adults living in the community).

**Results:** The key principles of co-creation are presented in four stages: Planning (what is the purpose of the co-creation; and who should be involved?); Conducting (what activities can be used during co-creation; and how to ensure buy-in and commitment?); Evaluating (how do we know the process and the outcome are valid and effective?) and Reporting (how to report the findings?). Three models are proposed to show how co-created solutions can be scaled up to a population level.

**Conclusions:** These recommendations aim to help the co-creation of public health interventions by providing a framework and governance to guide the process.

**Abstract:**

**Background:**

Due to the chronic disease burden on society, there is a need for preventive public health interventions to stimulate society towards a healthier lifestyle. To deal with the complex variability between individual lifestyles and settings, collaborating with end-users to develop interventions tailored to their unique circumstances has been suggested as a potential way to improve effectiveness and adherence. Co-creation of public health interventions using participatory methodologies has shown promise but lacks a framework to make this process systematic. The aim of this paper was to identify and set key principles and recommendations for systematically applying participatory methodologies to co-create and evaluate public health interventions.

**Methods:**

These principles and recommendations were derived using an iterative reflection process, combining key learning from published literature in addition to critical reflection on three case studies conducted by research groups in three European institutions, all of whom have expertise in co-creating public health interventions using different participatory methodologies.

**Results:**

Key principles and recommendations for using participatory methodologies in public health intervention co-creation are presented for the stages of: Planning (framing the aim of the study and identifying the appropriate sampling strategy); Conducting (defining the procedure, in addition to manifesting ownership); Evaluating (the process and the effectiveness) and Reporting (providing guidelines to report the findings). Three scaling models are proposed to demonstrate how to scale locally developed interventions to a population level.

**Conclusions:**

These recommendations aim to facilitate public health intervention co-creation and evaluation utilising participatory methodologies by ensuring the process is systematic and reproducible.

## Background

There is increased pressure on society to deal with a rising chronic disease burden [1]. The majority of this burden is attributable to modifiable lifestyle factors [2], for example poor nutrition [3] and low physical activity levels [4]. Therefore, there is a need for effective preventive public health interventions [5] and promotion to encourage individuals and groups of society towards a healthier lifestyle [6].

Current one size fits all interventions and those designed using a top-down approach have had limited success, potentially due to the complex influence of factors varying between individuals and settings [7]. Such complex public health issues have been labelled as “wicked” problems, which due to their heterogeneity, are difficult to fully understand [8]. One way to address these complex public health issues and to potentially deliver more effective and sustainable solutions is by tailoring interventions (defined as implementing strategies for individual or group needs in specific settings [9]) and developing localised solutions [10]. It has been suggested that, in order to ensure that interventions are tailored to the target end-users and settings, end-users and other non-academic stakeholders (such as peers or family) should collaborate with academic researchers to co-create interventions [11]. Despite an increasing variety of fields and theoretical perspectives that utilise co-creation [12], here we define co-creation as “collaborative public health intervention development by academics working alongside other stakeholders”.

Involving end-users and stakeholders in the co-creation of public health interventions and health promotion campaigns is increasingly advocated by funding [13] and governing bodies [14] as a more efficient solution to achieving positive societal changes. Engaging end-users in the development and design of products and services has a long history in economics [15], marketing [16] and business [17], to make products more marketable, appealing and to increase consumer loyalty [18]. The extent of end-user engagement is on a continuum ranging from: obtaining end-user feedback on a product designed by an expert designer, via co-creation approaches involving all actors on an equal contribution of knowledge throughout the development process, to meta-design which is initiated and controlled solely by end-users [19]. The characteristics of public health intervention development when applied to this continuum are visible in Table 1.

**Table 1:** Characteristics of participatory approaches applied to public health intervention development

Design	Traditional top-down	Co-creation	Meta-design
Decision Makers	Academic Researchers	Academic researchers & other stakeholders	End-users
End-user involvement	Co-option	Co-learning	Collective Action
Intervention Tailoring	One size fits all	Tailored to end-user by collaboration between co-creators	Tailored to end-user by end-user
Evidence Base	Large	Emerging	Limited

Traditionally, public health interventions are predominantly developed using a top-down approach. These are characterised by having a large evidence base and are often based on behaviour change theories thought to be applicable to all. However these traditional public health interventions do not involve end-users in their development. By comparison, utilising end-users in the co-creation of public health interventions is thought to increase adherence and effectiveness due to empowering end-users [20] to develop outcomes tailored to their circumstances [21]. Indeed, while few studies have yet to investigate the effectiveness of co-created interventions (i.e. do they result in positive change of the targeted public health problem), some report that these are more effective than one size fits all programmes [22] and lead to increased patient satisfaction [23] and a higher quality of service provision [24].

There are three groups of actors who may form the co-creation group (Table 2). In Table 2 the potential groups of actors (the stakeholder groups who engage in the process) are illustrated in an example case of co-creating a public health intervention for school-children.

**Table 2:** Potential groups of actors involved in the co-creation process (Example: intervention for school-children)

Group	Description	Example	Expertise
End-users	The group of people or population that is the target of the intervention	School-children	Provide insight of their specific needs
Stakeholders	The group of people who are interested or involved in the implementation of the intervention but who may or may not be the end-users. May also be referred to collectively as “non-academic stakeholders”	School-children Parents of school-children Teachers School directors Local authorities Educational bodies	Provide insight on intervention development and implementation from their perspective and experience.
Academic Researchers	Individuals who, in a traditional public health model, conduct the research	University researchers Public health practitioners	Provide evidence from current research

The co-creation process can be conducted by utilising one of a number of participatory methodologies, such as: Action Research (AR [25]), Participatory Action Research (PAR [20]) and Participatory Appreciative Action and Reflection (PAAR [26]), however this is not an exhaustive list. Recently, a significant body of work is emerging from social sciences under the broad heading of “Participatory Health Research” (PHR) [27], which was created to unite and increase the rigor of a variety of participatory methodologies. However, proponents of this approach seek to define PHR as a research paradigm rather than a methodology and advocate that PHR provide locally situated solutions. Consequently, less attention has been paid to co-creation governance and the scaling of co-created products to serve entire populations and large scale public health issues. Currently there is no precise or systematic framework to plan or develop co-created public health interventions and evaluate their effectiveness and impact [28]. In addition, there is increasing pressure from sponsors of research projects to demonstrate accountability through predefined performance indicators [28], therefore there is a need to embed co-created solutions into scientifically sound quantitative evaluation. Crucial concepts required in intervention research that PHR does not currently consider include: 1) developing a generalised protocol to ensure that the process of co-creating interventions is systematic and reproducible; 2) the formal testing of the effectiveness of co-created, locally developed interventions; 3) the creation of conceptual and pragmatic principles for scaling up locally developed interventions to address public health problems at a population level. Nevertheless, this gap can be addressed by merging the key elements of PHR within a classical intervention research paradigm.

Whilst we recognise that participatory methodologies have also demonstrated effectiveness in understanding key elements for intervention development, such as the determinants of health behaviours [29], this paper focuses on the systematic application of participatory methodologies in the co-creation and evaluation of public health interventions. Therefore, the aim of this paper was to detail principles and recommendations for the application and evaluation of public health intervention co-creation utilising participatory methodologies, and to provide models for scaling locally-developed solutions to a population level.

## Method

**Fig. 1:**
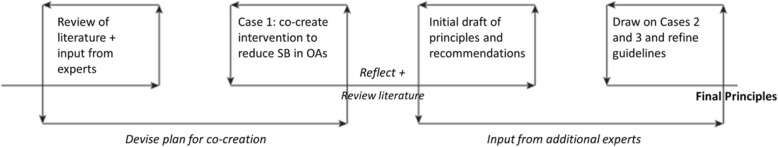
Iterative derivation of co-creation principles. SB = sedentary behaviour; OAs = older adults

The framework, principles and recommendations were developed using an action research approach [25, 30]. The multidisciplinary team worked through recursive learning cycles until full consensus about the framework and principles were achieved. The action research is based on the work carried out during the co-creation of three public health interventions (Fig. 1). These case studies originate from three European institutions (Glasgow Caledonian University, Scotland; Umea University, Sweden; Ghent University, Belgium):

### Case 1

The GrandStand Research Group created in Glasgow Caledonian University, Scotland (http://www.gcu.ac.uk/hls/co-creation/grandstand/). This group consisted of 11 community-dwelling older adults (mean age = 74 years) and four university researchers, who co-created an intervention with end-users to reduce sedentary behaviour in older adults [31].

### Case 2

The Safe Step project created in Umea University, Sweden. Here, 18 older adults (mean age = 74 years) worked with seven researchers in physiotherapy, informatics and computing science to collaboratively design an engaging exercise programme for strength and balance to prevent accident falls in older adults [32].

### Case 3

The Teenage Girls on the Move project from Ghent University, Belgium, in which three working groups across three secondary schools of lower educated adolescent girls (mean age = 16 years) each worked with a university researcher to co-create an intervention to promote physical activity.

These were a sample of convenience selected because of the variety of context and population they covered. Authors of each of these case studies were invited to take part in the action research to develop this framework and guidelines, in addition to others who have utilised co-creation to understand important elements for intervention development.

The reflective cycles consisted of analysis of field notes and reflective writing undertaken in each of the case studies, in addition to data gathered from participants during process evaluation conducted across sites. Reflection has been highlighted as a key learning process across a range of participatory methodologies, including in AR [33], PAR [34] and PAAR [26] and was utilised here to understand what elements contributed to the success of these projects. An initial framework and set of principles was developed by the lead author using previous examples of scientific framework development such as Cochrane PICO as guidance. This framework was then iteratively refined through evaluation of the data and experience of authors, with local co-creators also informing the process. Further, each team conducted thought experiments, guided by scenarios involving different contexts, populations and health behaviours, to determine how the framework and principles could be implemented to guide and improve the scientific rigour of future public health intervention co-creation. Throughout each iteration, authors inputted data and experiences to the lead author who synthesised, updated and redistributed the framework and principles. This process was repeated until a full consensus was reached. In total, there were eight iterations, six conducted by electronic correspondence and two face to face meetings.

Finally, the framework was written up as manuscript and then circulated again to the authorship teamt to ensure an accurate version of events was documented.

## Results

Following this process, five key principles were agreed for co-creating public health interventions using participatory methodologies: framing the aim of the study; sampling; manifesting ownership; defining the procedure; and evaluating (the process and the intervention). These were structured into four sections to provide a systematic approach: Planning, Conducting, Evaluation and Reporting. In addition, three models for scaling locally-developed solutions to a population level were identified (distributed model, generalisable model and cascade model).

### Planning

When academic researchers are initially planning the co-creation, there are two main principles that require consideration: framing the aim of the study and identifying the appropriate sampling strategy. The importance of these principles and recommendations of how to manifest them, are provided in this section.

### Principle 1: Framing the aim of the study

There are two main purposes to framing the aim of the study. First, it is important to ensure transparency with the co-creators about the aim of the process. Secondly, conducting co-creation processes with a project aim that is too broad has been highlighted as the reason many participatory projects are unsuccessful [28]. Therefore, clearly framing the aim of the study in a systematic way can help ensure the process generates scientifically trustworthy evidence.

The Cochrane (PICO) process, signifying the patient or problem (P), intervention (I), comparison (C) and outcome (O) [35], is a good example of enabling a clear identification of the important information required to frame the problem, allowing the problem to be defined in one sentence. This has lifted the review of literature process from a loose procedure to a more structured and systematic scientific enquiry, now regarded as one of the most thorough. Using this PICO framework as a guide, it is proposed here that a **PRODUCES** framework (**PR**oblem, **O**bjective, **D**esign, (end-) **U**sers, **C**o-creators, **E**valuation, **S**calability) could be adopted to facilitate a systematic approach when utilising participatory methodologies in co-creation.

#### **Pr**oblem

*What is the reason for the process?* A problem here is defined as an investigation of a fact or result [36] and where participatory methodologies are used to co-create a solution. Whilst in PHR, the focus could be on any problem, for public health intervention development, the problem should be narrowed to a specified health behaviour and population.

#### **O**bjective

*What is the aim of the process?* Many problems are complex and multifactorial, so when framing the aim of the study, it is important to define what part of the problem the process will address. Accidental falls, for example, are complex and many interventions may be effective to address this problem, such as exercise, adapting the environment, adjusting medications or providing information. Therefore, when framing the aim of the study, it is important to express the specific objective of the process, i.e. the *WHAT*. However the non-academic co-creators will influence what the intervention actually looks like, i.e. the *HOW*. When addressing a complex problem, end-users and other stakeholders may collaborate with academic researchers to define the objective.

#### **D**esign

*What specific participatory methodology is used for co-creation?* It is important to identify the specific participatory methodology that will be used to guide the co-creation, as this will directly impact how the process is carried out, scalability and potential inference that can be derived.

#### (End-) **U**sers

*Who will use the co-created intervention?* Defining the end-users is simply to consider which specific population the intervention is being designed for. The end-user group can be defined specifically or broadly, however this will impact the flexibility of the final outcome. For example, defining the end-user group as “older adults” will mean co-creating an intervention which has to cater for many sub-groups of this population, compared with defining the end-user group as “ambulatory, community-dwelling older adults”. End-user characteristics to consider may be: age, gender, physical condition, medical history, socioeconomic status and ethnicity. A representative sample from the end-user group should be recruited as co-creators during the development process. This may include sampling across age, gender, severity of condition (if appropriate) and socio-economic status to create a heterogeneous group with diverse experiences.

#### **C**o-creators

*Who is engaging in the process?* It is important to identify who the co-creators are as, unlike traditional intervention development, this will involve a combination of service providers and end-users [37]. When co-creating a public health intervention, the groups of actors who engage in this process may include academic researchers (who may also assume the role of facilitating the process) and a combination of end-users and relevant stakeholders (dependent on the population group). Strategies for manifesting co-ownership to ensure equal contribution and sharing of expertise between these groups of actors are discussed in the Conducting section.

#### **E**valuation

*How is success measured?* When co-creating a public health intervention, evaluation may take two forms. Firstly, the co-creation process may be evaluated by considering factors including co-creator satisfaction and ensuring the developed intervention is representative of co-creators’ opinions. Secondly, the effectiveness of the co-created intervention can be evaluated by embedding the outcome into a clinical trial. More in-depth information regarding evaluation is available in the evaluation section.

#### **S**calability

*How can the solution be scaled to a population level?* If appropriate, it is important to consider how the developed solution can be used to target a wider population to achieve greater impact. This can be achieved by utilising one of the three scaling models discussed later.

Table 3 shows examples how the PRODUCES method has been used to frame the aim of the three case studies highlighted here.

**Table 3:** Framing case studies using the PRODUCES method

PRODUCES	Case 1	Case 2	Case 3
**Pr**oblem	Older adults are too sedentary and spend long periods sitting	Accidental falls causes individual suffering and substantial costs to society	Adolescent girls have low levels of physical activity. Many interventions fail to induce an effect on girls’ physical activity level, often because this target group is difficult to reach and less likely to engage in interventions
**O**bjective	To design an intervention that will help older adults break up long sitting periods	To design a mobile application that will enable seniors to create and adhere to an individually tailored strength and balance exercise programme	To design an intervention to promote physical activity
**D**esign	PAAR	PAAR	PAR
(end-) **U**sers	Community-dwelling older adults over 65 years of age	Community-dwelling older adults over 70 years of age	10th grade lower educated adolescent girls
**C**o-creators	University researchers and community-dwelling older adults	University researchers and community-dwelling older adults	University researchers and lower educated adolescent girls
**E**valuation	Process evaluation	Process and effect evaluation	Process and effect evaluation
**S**calability	Generalisable model	Generalisable model	Distributed model

*PAR* Participatory Action Research, *PAAR* Participatory Appreciative Action and Reflection

### Principle 2: Sampling

Whilst the purpose of sampling has traditionally been to construct statistical inferences to the population derived from the sample [38, 39], this may not be appropriate for co-creation. The sampling process for recruiting co-creators is dual purpose: 1) ensure a representative sample of end-users are recruited as co-creators so the co-created outcome can be utilised by that group or scaled to a population level; 2) ensure there is representation of all necessary expertise from relevant stakeholder groups.

#### Approaches to sampling co-creators

Whilst end-user co-creators should always be representative of the end-user population, the representation of relevant stakeholder groups is dynamic and may involve recruiting additional stakeholders throughout the process, known as opportunistic sampling [40]. Convenience sampling is commonly adopted when utilising participatory methodologies [41] and is valuable to ensure the recruited co-creators are committed and will actively engage in the process. To ensure a representative sample of end-user and relevant stakeholder co-creators are recruited, purposeful sampling may be advantageous [42]. This may include stratified sampling across characteristics of interest to create a heterogeneous group with diverse experiences. Finding end-users who cover the spectrum of perspectives, including typical and extreme cases, is known as maximum variation sampling [43]. Prioritising potential co-creators based upon their motivations for participation has also been cited previously as factors to consider when sampling [44].

#### Co-creator sample size

Qualitative research does not have existing rules regarding recommended sample sizes [45], however recommendations have been made to recruit cohorts of between 6 and 12 participants for focus group studies [46]. In co-creation, the procedure is similar to that of focus groups, as high quality interactive discussions between the co-creators is pivotal for the process to be successful [18]. Additionally, recruiting the upper range of this recommendation [46] allows for the group to be divided into smaller groups (such as previous research using groups of seven individuals [47]) for certain interactive activities (examples available in the procedural section). Considering these factors, a recommendation of 10–12 co-creators is advised, which may also account for dropouts due to the process being conducted over a series of meetings. 

### Conducting

This section discusses principles that should be considered when conducting the co-creation process: manifesting ownership and defining the procedure (distinguishing between important procedural components and procedural methods).

### Principle 3: Manifesting ownership

Ownership is the state, right or act of possessing something [36]. Ownership may improve creativity [48], practice and knowledge production [49] by providing co-creators with a sense of belonging [50]. The outcome of the co-creation process does not have to be tangible for ownership to be manifested [51], for example development of a mobile phone application. Ownership has been identified as a key [52] yet understated [53] dimension of co-creation, therefore recommendations are provided in line with its definition to help facilitate its manifestation.

#### State of ownership

This can be achieved by branding the group of co-creators and setting out the status of each co-creator at the beginning, such as affirming that all co-creators have equal standing in the group. Ownership may be acquired incrementally over several workshops through an empowerment process [19] of facilitating openness and perceived control of the process.

#### Right of ownership

The co-creators have the right to the knowledge required in order to exercise meaningful participation [54], such as collectively defining the specific aims necessary to address in order to achieve the overall objective [55]. End-user co-creators have the right to receive data from academic co-creators regarding the current evidence base for the health behaviour being targeted to inform their decision-making. End-user co-creators also have the right to be trusted by the academic researchers and require power-sharing governance arrangements in order to exercise empowered participation [28].

#### Act of ownership

Once co-creators perceive a state and right of ownership, they have the responsibility to act upon that ownership. For example, as ownership occurs from self-investment [56], co-creators have the responsibility to invest themselves in the process and may be required to provide input at each stage. Examples of co-creators acting on ownership may include peer reviewing autonomous tasks, which will help ensure that 1) the process is participative and semi-democratic [53] and 2) the quality of contribution, and therefore the integrity of the process, is addressed.

### Principle 4: Defining the procedure

This section provides recommendations and examples of procedural components and methods that can be used during the co-creation of a public health intervention. These are not necessarily sequential or mandatory, however are likely to facilitate the co-creation process if adopted.

#### Procedural component: Highlight purpose of the process

Once the aim of the study has been framed in the planning section, this must be clearly stated and agreed by the co-creators from the beginning of the process and used as a point of reference that is reiterated throughout [57]. Co-creators should contribute their ideas and knowledge to ensure the process is successful [58] and agreeing on the purpose of the process can ensure ideas are structured in a systematic way. However, identifying the overall aims of the process does not provide an accurate signal of progress [59], therefore short-term aims should also be acknowledged [60] to identify the timing of milestones which will be achieved over the process.

#### Procedural component: Highlight rules of participation

The purpose of this component is to emphasise the rights and responsibilities of the co-creators that closely link to the manifestation of ownership (previously discussed). For example, all co-creators have the right to have control [61] during the process and also to have intimate knowledge [51] of the problem. However, co-creators also have the responsibility to invest themselves [56] in the process and must provide their viewpoints [58] to deliver a fruitful outcome. These will be achieved by clearly agreeing, stating and identifying the status and role of each co-creator.

#### Procedural component: Up-skill co-creators

When developing a new public health intervention, two essential pieces of information are required: 1) the preferences and experience of the end-user (which initially, the end-users (and in some cases, other non-academic stakeholders) have the most knowledge of) and 2) potentially effective strategies (which initially, the academic researchers may have the most knowledge of) [58]. As co-creation should engage academic and non-academic stakeholders on an even distribution of power and knowledge, it is necessary for all co-creators to share their knowledge to create equality. For example, if co-creating an Exergame for children, three groups of actors who are required to provide their expertise include: academic researchers (expertise on the health problem and behaviour change techniques), children (expertise on their own needs and preferences) and computer game developers (expertise on potential game play techniques that can be used). Up-skilling can increase the capacity and capability of the co-creators [62] and potentially result in the development of more innovative and meaningful solutions [19].

One method to up-skill non-academic co-creators is to review evidence from previous research (such as previous interventions that were effective in improving a specific health behaviour in a similar population). This is an example of resource sharing that is a valuable element in co-creation processes [63] and can be used as a foundation on which to build discussions regarding the development of the intervention [57]. Whilst reviewing previous evidence can provide a scaffold that supports co-creators’ creativity [15], it is important to reiterate that the information being conveyed is not the entirety of the reality. Reviewing research evidence should not become a dogma and instead, should be used in conjunction with open-ended discussions [15] and evidence provided by non-academic co-creators to ensure end-users’ needs are met [18]. It is important to highlight that mutual learning will occur across stakeholders and academic co-creators can expect to be up-skilled in numerous areas, including the needs and preferences of end-users.

#### Procedural component: Prototyping (or pilot testing)

Prototyping involves developing a conceptual product based on the information gathered during the co-creation process [57]. Prototyping has been highlighted as a valuable strategy towards bringing the creation of ideas together [64] and allows for co-creators to visualise their thoughts [65] and suggest improvements that can be made [57] to enhance the intervention [66]. The information included in the prototype should be provided as early as possible during the process, which will ensure that co-creators have a similar understanding as to the concept of the final intervention [67]. This highlights the importance of the up-skilling phase, so the co-creators have the necessary skills (such as learning about concepts of research methods) to develop and test the prototype.

#### Procedural component: Iterative process

**Fig. 2:**
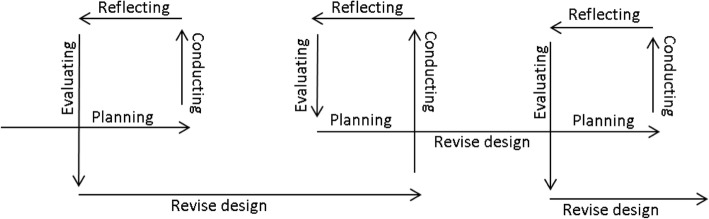
Iterative co-creation process

Iteration, which is defined as a cyclical process, includes planning, conducting, reflection, evaluation and refining a process or a product (Fig. 2) [36] and is a central dimension to both motivation and learning in co-creation [68]. The process is not linear but an act of repetition, whereby each repetition or “iteration” is used as the starting point for the next iteration with the overall aim of approaching a desired goal through learning and reflection.

Iteration during co-creation may occur on several levels to stimulate the process and to incrementally refine the designed prototype or intervention. Examples of iterative procedures are: 1) Revisit the main findings from the previous meeting in the form of a compiled report, which can be used as a starting point for discussion in the following meeting. Setting the stage at the beginning of each meeting is important [57] to be clear of how the previous discussions help the co-creators reach the overall goal of the process; 2) Throughout the process, certain discussion points may be reiterated to help the co-creators identify what the core components of the intervention should include whilst various cycles of the process are completed [69]; 3) Gradually developing and refining several versions of a prototype [57].

#### Procedural method: Structure

Previous literature recommends the co-creation process to last between 3 and 4 full days, the equivalent of between 18 and 28 h [57]. In Case 1 (working with older adults), the process comprised of 10 meetings lasting around two hours in addition to tasks outside the workshops, similar to previous work [70]. By comparison, the meetings in Case 3 (working with adolescents) were restricted by the school lunch break, however academics had additional contact with end-user co-creators through social media, therefore the process is not necessarily limited to organised meetings.

The structure of each meeting may begin by reiterating the purpose of the process. Additionally, a schedule can be set out; however this must be adaptable depending on how the process evolves and therefore any change in the direction of the design. For example, the further along the participatory continuum the project is, the more involvement there will be from co-creators (Table 1) and therefore, the structure must be flexible but should still follow a protocol guided by PRODUCES and focus on answering the aim of the project. Previous studies have ensured meetings had a very flexible structure led by the end-user co-creators to ensure their creativity was not curtailed [29]. Further, the aims and objectives should be identified in each meeting, helping to place the present meeting within the overall context of the process.

#### Procedural method: Interactive techniques during the meeting

High-quality and creative interactions are essential towards a valuable co-creation process [18], for example interactions between co-creators, interactions with the prototype and interactions with the meeting tasks. Without this deep interaction between co-creators, new ideas and products cannot be materialised [67]. Table 4 details some examples of interactive methods that can be used and have been used in these case studies; however this is not an exhaustive list.

**Table 4:** Examples and definitions of interactive techniques that can be used during co-creation

Interactive Technique	Definition
Brainstorming [89]	Compiling a spontaneous list of ideas, as a group, to develop a solution for a particular problem
Scenarios [57]	Identifying the needs of end-users in different contexts by developing situations which may be problematic when addressing the purpose of the co-creation
Personas [90]	Focus on the end-user and the qualities and preferences they possess which should be considered when developing an intervention. Has been used previously to co-create an Exergame for older adults with a history of falls [91]

#### Procedural method: Fieldwork tasks outside the meeting

Fieldwork outside of the meetings can be beneficial to gain a deeper understanding of the health problem being addressed. For example, fieldwork tasks allow for co-creators to identify if there are any environmental factors that may impact the performance of an intervention outside the setting in which it is created [71]. Therefore, environmental observation is likely to inform the intervention design, for example playground characteristics which may be important for school-children when trying to increase physical activity [29]. Table 5 details examples of fieldwork tasks that can be conducted between meetings to collect additional data, however this list is not exhaustive.

**Table 5:** Examples and definitions of fieldwork tasks that can be used outside the co-creation process

Fieldwork task	Definition
Field testing of prototype	Provide co-creators with an example of what the final intervention may be like. Allows end-users to use the prototype in their typical real world setting and identify how it could be improved [57] and will inform the implementation of the intervention itself.
Media portrayal of the co-creation purpose	Gather printed media images, such as from newspapers and magazines, which convey the purpose of the process [31]. Using visual aids as discussion tools in the following meeting has been identified as an effective tool to enhance the effectiveness of co-creators [92].
Interactions with non-academic stakeholders outside the co-creation group	Co-creators informally discussing process topics with non-co-creator stakeholders. Can provide a fresh perspective to the topic and enhance ideas [69] and is an informal form of snowball sampling that may increase the generalisability of findings.

### Evaluation

The purpose of evaluation is to establish the quality of an intervention [72] and may occur in two forms: 1) evaluation of the process (exploring factors such as co-creator satisfaction and whether the designed intervention is a reflection of their needs and preferences); 2) evaluation of the co-created intervention (formal testing of its effectiveness to examine whether the intervention actually results in a positive change in the targeted public health problem).

### Principle 5a: Evaluating (the co-creation process)

When co-creating an intervention utilising participatory methodologies, evaluation of the process is a key part of the procedure [73]. As co-creation is an iterative process, evaluation may be embedded throughout the process to ensure the process results in an outcome that is representative of co-creators’ opinions and suitable, tailored and valid for end-users.

#### Validity of the outcome and the process

Member checking [74] can be used throughout the process to increase the rigour of findings [75]. This will involve the co-creators reflecting on their previous discussions and can therefore be used as a tool for continual learning throughout the process [76]. Reflection occurring by the co-creators interacting with each other can help uncover both strengths and weaknesses in thinking and therefore improve the developed intervention [77]. Other strategies advocated to validate the findings include respondent validation [78], whereby a summary of key intervention components previously discussed are presented to co-creators and subsequently refined [75]. These strategies may be particularly beneficial to use on prototypes developed during the process, including a concept of the final outcome, in order to refine findings. Here, it may also be important for the co-creators to evaluate how closely the process adheres to the PRODUCES reporting guidelines highlighted later, for example how many recommendations they fulfilled and which they did not (for a detailed breakdown of PRODUCES, see the “Reporting” section).

#### Co-creator satisfaction and ownership

In Meta-design, evaluation criteria include assessing the co-creators perceived engagement and enjoyment of the process [19]. In co-creation, ownership may be measured by loyalty [69], for example assessing the retention / dropout rate of co-creators. Evaluation questionnaires may also be distributed to the co-creators [29] and elements that may be of interest to examine include: satisfaction of engaging in the process, feedback on the developed intervention [79] and perceived knowledge and skill development [80].

### Principle 5b: Evaluation (the effectiveness of the co-created intervention)

**Fig. 3:**
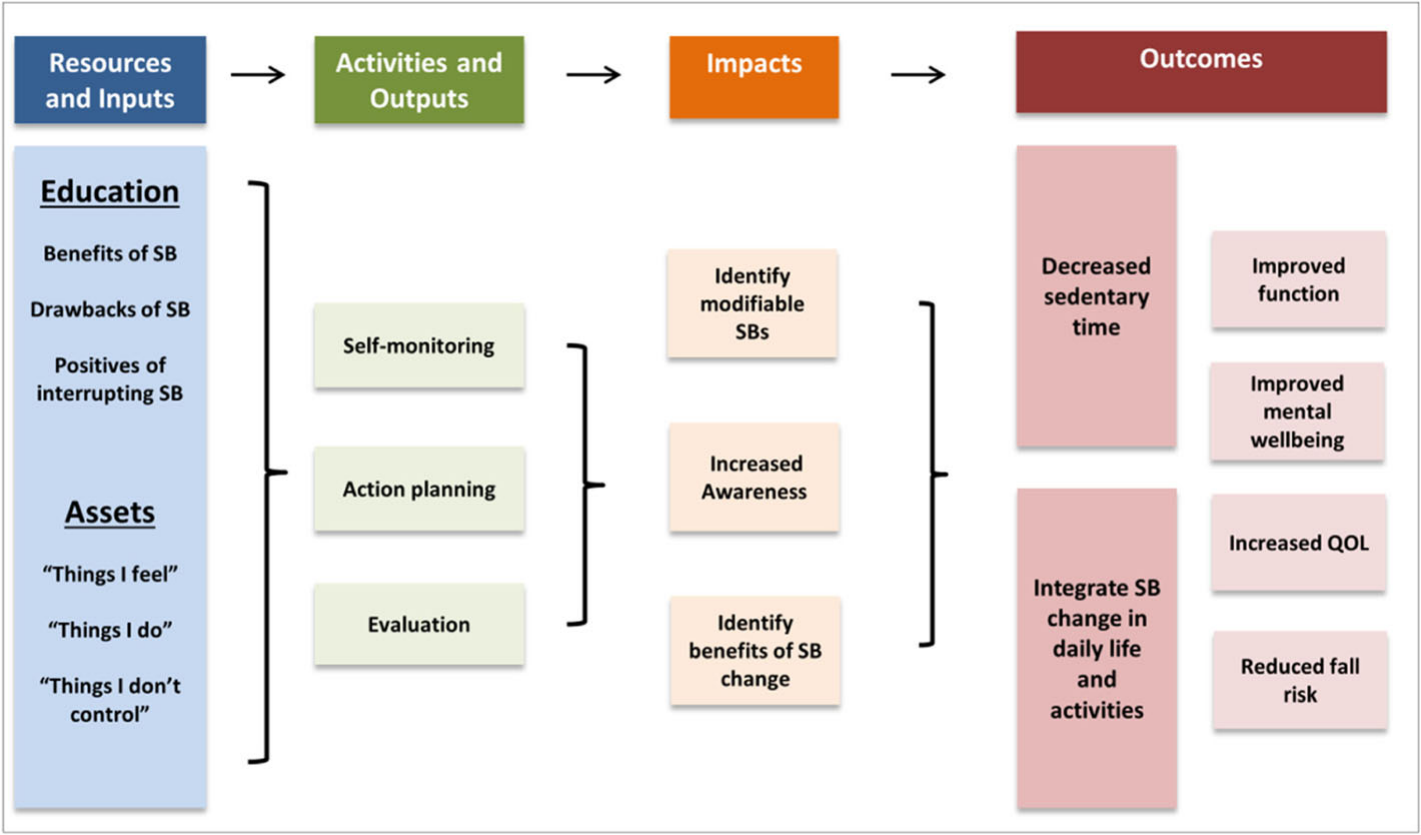
Logic model of Case 1 co-created intervention mechanism. QOL = quality of life; SB = sedentary behaviour

Before assessing whether the co-created intervention is effective, as outcomes will not necessarily be theory-driven, a logic model may be generated to help explain the potential mechanism of how the intervention works [81]. An example of the logic model generated for the co-created intervention in Case 1 is visible in Fig. 3, however it should be noted that the effect of this intervention has yet to be trialled [31].

Previous participatory projects have evaluated the effectiveness of interventions by measuring outcomes such as improving healthy eating, increasing physically activity [82] or improving immunisation rates [83]. However, ecological comparisons such as these do not control for other community-operating factors [83]. Therefore, we recommend that formal testing of an intervention involves embedding the co-created intervention into a formal positivist research framework. This may occur in two phases: 1) determine whether the co-created intervention is effective locally and 2) if it is effective locally, assessing its effectiveness at a population level. There are several possibilities to evaluate the effect of the intervention, for example by embedding the co-created intervention into a randomised controlled trial (RCT), compared to standard care or traditional top-down developed interventions. The design of this trial will depend on the research question, population and context. If effect is shown, this process can be scaled in several ways to derive population-level inference, including 1) conducting a nationwide RCT comparing co-created versus top-down interventions or 2) meta-analysing the findings from several localised RCTs. A process evaluation of the intervention may also be conducted, with key components including fidelity, proportion of reach, facilitators and barriers to implementation being areas of interest to explore [84].

### Reporting

In order for the co-creation process to be clearly understood, reproducible and systematic, we recommend completing a checklist in order to compare other co-created interventions to a set standard. Such checklists have previously been developed, including PRISMA [85] for reporting systematic reviews and meta-analyses, along with CONSORT [86] for reporting RCTs. Below is the checklist proposed and an example of how to complete it (Table 6).

**Table 6:** Checklist for reporting intervention co-creation

Section	Checklist Item	Case 1
Planning		
How was the aim of the study framed?	1) Use each element of the **PRODUCES** framework (**PR**oblem, **O**bjective, **D**esign, (end-) **U**sers, **C**o-creators, **E**valuation and **S**calability)	Utilising PAAR (**D**esign) to develop (**O**bjective) and test (**E**valuation), with academic researchers and older adults (**C**o-creators), a generalisable (**S**calability) intervention to reduce sedentary behaviour (**PRoblem**) in community-dwelling older adults (end-**U**sers).
Explain the sampling procedure	2) Explain the criteria used for sampling	Convenience sampling and maximum variation sampling. End-users were 65+ years of age, community-dwelling, able to ambulate independently, able to give informed consent, able to attend a minimum of 5 meetings.
	3) In what settings did sampling occur?	End-user co-creators were recruited from university older adult database
	4) How many individuals engaged as co-creators (academic / non-academic stakeholders)?	Four university researchers and 11 community-dwelling older adults
	5) Describe the co-creators (demographics / groups / other characteristics of interest).	Of the end-user co-creators, 11 participants (5 men), average age = 74 years. Average medications = 5.
Conducting		
How was ownership manifested?	6) Explain the methods used to manifest ownership (for example, branding the group, identifying the rights and responsibilities of the group)	Co-creators branded as GrandStand Research Group. Co-creators provided with t-shirts, lab books, bags and pens with GrandStand logo. All co-creators told of their right of equal status within the group and their responsibility to contribute their ideas.
Procedure Components:	7) What level of participation was there from the co-creators?	Academic researchers and end-users strove to have equal participation. All co-creators asked for their input on each discussion point.
	8) How was the overall aim presented?	Overall aim highlighted at the beginning of the process and beginning of each workshop.
	9) How was the purpose of each meeting presented?	Purpose of each meeting identified at the beginning of the meeting.
	10) What were the rules and responsibilities of participation agreed upon?	Individuals told of their right of equal status within the group and to contribute their ideas.
Procedure Methods:	11) In which areas did the co-creators require up-skilling?	End-users were up-skilled regarding behaviour change theory and research methods. Academic researchers were upskilled regarding older adults’ reasons and preferences for interrupting SB.
	12) What previous evidence was reviewed, and how?	Presentations of the context of older adults’ SB [93], behavioural assets which can be used to interrupt sedentary periods [94] and behaviour change theories.
	13) If a prototype was developed, describe the prototype and the prototyping process	Full intervention prototype created from several key components which were individually prototyped, tested by co-creators and then refined.
	14) Describe the frequency and duration of meetings	Meetings occurred every 10–14 days and lasted approximately 2 h.
	15) Give examples of interactive techniques or methods used	Scenarios (eg. where it may be easy / difficult to break sedentary behaviour); Brainstorming (eg. how to best categorise older adults’ assets)
	16) Give examples of fieldwork techniques or methods used	Testing created prototypes with end-users not involved with the process.
	17) Give examples of how iteration occurred during the process	Prototypes were initially developed, tested externally and after discussions, refined and then tested again.
Evaluation		
Process	18) Explain how co-creator satisfaction and contribution evaluated (for example reporting on attendance rates, questionnaires, interviews).	Retention rates measured (100% retention, 0% dropout).
	How are results reported back to stakeholders and the public?	Written reports were developed explaining the results of the process and findings were disseminated at national conferences.
Outcome	19) Explain how the validity of the outcome and the process were evaluated (for example, face validation, member checking).	Face validation and member checking occurred throughout, including each developed prototype component and a summary of the information gathered from the previous meeting.
	20) Explain plans for formal testing of the effectiveness/scalability of the co-created outcome	Plan to embed the intervention into a multi-centre RCT vs. a top-down, theory-driven intervention and standard care (control group) to assess the effectiveness of the intervention.
	21) Explain outcome of evaluation (if tested)	If step 20 is deployed, outcomes which will be measured will include: changes in sedentary time, changes in sedentary time fragmentation, participants’ experience of using the intervention and effect on function (noted as important by the end-user co-creators.

*SB* Sedentary behaviour, *RCT* Randomised controlled trial

### Scaling

**Fig. 4:**
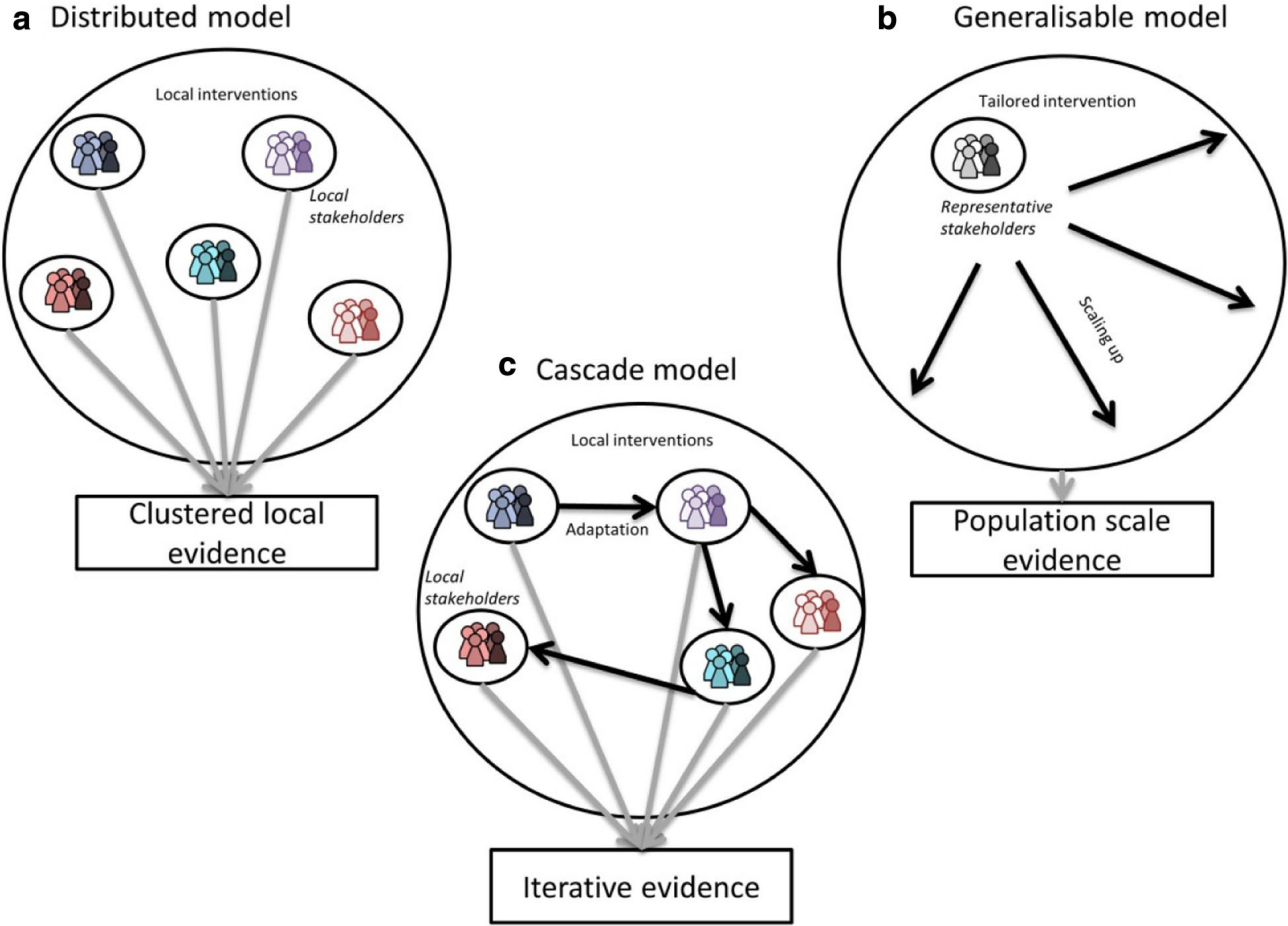
Models for scaling locally co-created public health interventions using participatory methodologies: **a** distributed model; **b** generalisable model; **c** cascade model

By nature, co-created solutions are localised [28], therefore to address public health issues, there is the need for strategies to scales these interventions to a wider population level. As such, three models are described here to achieve this scaling (Fig. 4). In the distributed model, the design and implementation of each intervention is developed and initiated locally in collaboration with or by local actors specifically for this local group and setting (Fig. 4a). In this model, local solutions from multiple locations are developed independently and clustered in order to reach a larger population. Here, the actual co-creation process can be used as an intervention itself to assess whether engaging in the development process has influenced the end-users’ targeted health behaviour. The generalisable model is to develop, in collaboration with a sample of stakeholders and representative end-users of a larger population, a tailored intervention that can be scaled and implemented in a larger group (Fig. 4b). In the cascade model (Fig. 4c), one local intervention is designed and implemented locally in collaboration with or by local stakeholders and end-users specifically for this local group and settings. This solution is then transported and adapted in collaboration with or by a new group of local stakeholders and end-users for the same purpose, in a different setting.

## Discussion

In this paper, key principles and recommendations for the application of participatory methodologies in the co-creation and evaluation of public health interventions are delineated to provide a systematic framework for planning, conducting, evaluating and reporting findings. In the future, academic researchers can plan their investigation using **PRODUCES** to clearly define which **PRo**blem (health behaviour issue) they wish to address; the **O**bjective of the process; which participatory methodology **D**esign they will use; who are the end-**U**sers; who will engage as **C**o-creators; how the intervention will be **E**valuated and which model to adopt to ensure its **S**calability. To ensure true stakeholder engagement and scientific rigor, the process can be guided by the principles and recommendations highlighted. Finally, to enable scientific synthesis, academic researchers can now follow reporting guidelines.

Some of the principles and recommendations highlighted here have been reinforced in two recently conducted literature reviews on co-creation in health services [28] and participatory research in health research [87]. For example, Greenhalgh et al. [28] highlighted the importance of a systems perspective, citing that outcomes are not predictable in advance on the assumption that the process is nonlinear and is adapted locally. Here, the co-creation process was highlighted as iterative (Fig. 2), whereby particular discussion points and intervention elements may be revisited over several meetings in order to refine and enhance them. Additionally, the local adaptation assumption highlighted by Greenhalgh et al. [28] is presented in the cascade model (Fig. 4c), which signifies that locally co-created solutions can be transported and adapted to different settings to ensure local relevance. Further principles that mirror this review include emphasis on framing the program (similar to our “framing the aim of the study” principle) and governance and facilitation arrangements (similar to our recommendation to highlight the rules of participation) [28]. One key principle highlighted by Jagosh et al. [87] was partnership synergy, with authors explaining that combining actors skills and resources was an integral components towards creating a new and valuable outcome. Here, reviewing previous research was identified as one example of resource sharing which should be embedded into the procedural component of the co-creation process. These similarities shown with the literature reviews cited further reinforces the principles and recommendations identified here and their applicability to other public health contexts.

Examples of other principles that did not emerge here include managing conflict, with both previous studies suggesting that conflict between co-creators can be an engaging and positive force which is an integral component of establishing rapport and trust [28, 87]. One reason hypothesised for this finding was due to involving government and community partners in co-creation processes, which could result in competing interests arising and from which, conflict can occur [28]. In the case studies presented here, there were only two groups of actors (academic researchers and end-users), and conflict did not emerge as a tool to enhance co-creator relationships, therefore relevant experience could not be drawn to derive practical recommendations for future studies regarding how to effectively utilise conflict.

One of the opportunities offered by utilising co-creation may be a more efficient and cost-effective intervention design process. As lifestyle is influenced by a complex web of factors which vary between individuals and settings [7], the number of parameters to consider when developing an optimum and tailored intervention are theoretically infinite. Therefore, the current top-down approach which academic researchers implement to understand which combination of these parameters are the most effective is perhaps not the most time efficient or financially viable option. Adopting a distributed participatory model, despite only emerging recently as a paradigm (Fig. 4a) may be one effective strategy to speed up this process by simultaneously developing and testing multiple versions of an intervention with the same function.

For academic researchers wishing to utilise participatory methodologies to co-create public health interventions, they must acknowledge the necessary changes required in scientific practice to ensure the process is conducted effectively. Participatory methodologies redefine the role of the academic researcher, from the focal decision-maker towards an equal distribution of power between academic and non-academic stakeholders. As academics may traditionally begin the research process from the perspective that they are central to intervention development [88], there may be reluctance to relinquish the power these decision makers traditionally possess. However, academic researchers who do not fully accept or implement the governance associated with co-creation may endanger the veracity and effectiveness of the process.

There are some limitations which should be acknowledged. As discussed previously, this paper reflected on case studies engaging only two groups of actors (academic researchers and end-users). As a result, there may be other principles beneficial to implement in the co-creation process, particularly when engaging multiple groups of actors, which have not been cited here. However, given the dearth of information regarding best practice when co-creating public health interventions, utilising two actor groups was agreed as a pragmatic first step on which to derive key learning. As some of the case studies included a convenience sample of non-academic stakeholders, the findings reflected on may have been influenced by bias due to the sample not being fully representative of the end-user groups.

## Conclusion

This paper provides detailed principles and recommendations for the planning, conducting, evaluation and reporting of public health interventions developed utilising participatory methodologies. These can be used as a framework to ensure co-creation is conducted in a systematic and reproducible way which may allow for more generalised principles to be detailed in the future from different projects. Future work should aim to apply this framework to co-create local public health interventions and subsequently scale these to the population level using the proposed scaling models.

## Abbreviations

AR: Action research
PAAR: Participatory appreciative action and reflection
PAR: Participatory action research
PHR: Participatory health research
PRODUCES: PROblem, Design, (end)-users, Co-creators, Evaluation, Scalability
